# Two-layer interrupted versus two-layer continuous sutures for preventing cesarean scar defect: a randomized controlled trial

**DOI:** 10.1186/s12884-025-07353-1

**Published:** 2025-03-07

**Authors:** Shunichiro Tsuji, Daisuke Katsura, Shinsuke Tokoro, Ayako Inatomi, Yuri Nobuta, Yutaka Yoneoka, Tsukuru Amano, Takashi Murakami

**Affiliations:** Department of Obstetrics and Gynecology, Shiga University of Medical Science, Seta Tsukinowa-cho, Otsu, Shiga 520-2192 Japan

**Keywords:** Cesarean scar defect, Cesarean scar disorder, Cesarean section, Continuous suture, Interrupted suture

## Abstract

**Background:**

Cesarean scar defects can lead to long-term complications, such as cesarean scar disorders, cesarean scar pregnancy, and the risk of uterine scar dehiscence and rupture in subsequent pregnancy. However, the optimal closure technique to prevent the development of cesarean scar defects (CSD) remains unclear. Therefore, this study aimed to explore whether two-layer interrupted versus two-layer continuous sutures could prevent the formation of CSD.

**Methods:**

A randomized controlled trial was conducted in a single university hospital in Japan. We recruited pregnant women with ≥ 20 primary or previous cesarean sections. Participants were randomly assigned to either a two-layer interrupted or a two-layer continuous suture group. Residual myometrial thickness (RMT) and the depth of CSD were measured using sonohysterography, 6–8 months post-cesarean section. In addition, the rate of severe CSD, defined as a loss of over 50% of the myometrium, was examined.

**Results:**

Of the 220 study participants, 43 dropped out; 89 in the interrupted group and 88 in the continuous group underwent sonohysterography. No significant difference in RMT was observed in the interrupted and continuous groups (median 8.1 [interquartile range, 6.2–9.9] mm and 7.9 [4.6–10.3] mm, respectively). However, the incidence of severe CSD in the interrupted group was significantly lower than that in the continuous group (2% versus 22%, *p* < 0.0001). Multivariate logistic regression analysis revealed that the factors contributing to developing severe CSD were interrupted suture (odds ratio [OR]: 0.04, 95% confidence interval [95%CI]: 0.006–0.281, *p* = 0.0011), the difference in myometrial thickness between the fundal and cervical sides at the center of the uterine wound before suturing (OR: 1.65, 95%CI: 1.144–2.367, *p* = 0.0072), and retroversion of the uterus at 6–8 months after cesarean section (OR: 3.42, 95%CI: 1.074–10.946, *p* = 0.0374).

**Conclusion:**

This study suggested that two-layer interrupted sutures are superior to two-layer continuous sutures in preventing the development of severe CSD.

**Trial registration:**

Clinical trial identification number: University Hospital Medical Information Network registration code, UMIN000040601. URL of the registration site: https://center6.umin.ac.jp/cgi-open-bin/ctr_e/ctr_view.cgi?recptno=R000046334.

**Supplementary Information:**

The online version contains supplementary material available at 10.1186/s12884-025-07353-1.

## Background

Although cesarean section (CS) is an essential surgical procedure to save the life of the mother and fetus, it can lead to a cesarean scar defect (CSD) due to the thinning of the residual myometrial thickness (RMT). In subsequent pregnancy, CSD may contribute to cesarean scar pregnancy and uterine scar dehiscence and rupture. Additionally, even if pregnancy does not occur after CS, CSD may result in various gynecological symptoms, such as postmenstrual spotting, dysmenorrhea, chronic pelvic pain, and secondary infertility. These symptoms are called cesarean scar disorders [1, 2]. Therefore, it is essential to establish a proper suturing procedure to prevent CSD.

Various studies have demonstrated surgical techniques to avoid thinning of the residual myometrium. Although no suturing technique has been established to avoid thinning of the myometrium, there have been some reports that two-layer sutures may be superior to single-layer sutures, and unlocked sutures are superior to locked sutures [3–6]. Cecio et al. demonstrated that a single-layer interrupted suture led to a smaller CSD than a single-layer continuous suture [7]. However, although two-layer interrupted and two-layer continuous sutures are commonly used in Japan, no reports have prospectively compared them.

CSD is an indentation of at least 2 mm on the myometrium at the site of the CS scar measured by transvaginal ultrasonography (TVU) [8], but not all cases of CSD result in gynecological symptoms. Zhou et al. reported that the mean RMT in the asymptomatic CSD group was significantly thicker than that in the symptomatic CSD group (5.4 mm versus 3.2 mm, respectively) [9]. Mohr-Sasson et al. demonstrated that RMT < 2.5 mm was significantly related to heavy menstrual bleeding and secondary infertility [10]. Therefore, preventing severe CSD is essential. Ofili-Yebovi et al. defined severe CSD as the loss of over 50% of the myometrium at the site of CS and demonstrated that the frequency of severe CSD was approximately 10% post-CS [11].

Herein, we compared two myometrial suturing techniques, two-layer interrupted and two-layer unlocked continuous sutures, and evaluated RMT and the incidence of severe CSD 6–8 months post-CS.

## Methods

### Study design and participants

The Ethics Committee of the Shiga University of Medical Science approved this randomized controlled trial (approval number C2019-322), which was conducted between May 2020 and October 2023 at the Shiga University of Medical Science in Japan. This study was registered at the University Hospital Medical Information Network center before performing the trial (registration code: UMIN000040601) and adhered to Consolidated Standards of Reporting Trials guidelines and the Declaration of Helsinki. Patient recruitment was conducted between June 2020 and April 2023 to evaluate RMT and the incidence of severe CSD 6–8 months post-CS. The inclusion criteria were age 20 years and CS performed at the Shiga University of Medical Science. The exclusion criteria were uterine malformations, uterine fibroids around the incision line of the uterus, those who underwent surgery between CS and CSD evaluation.

### Randomization

**Fig. 1:**
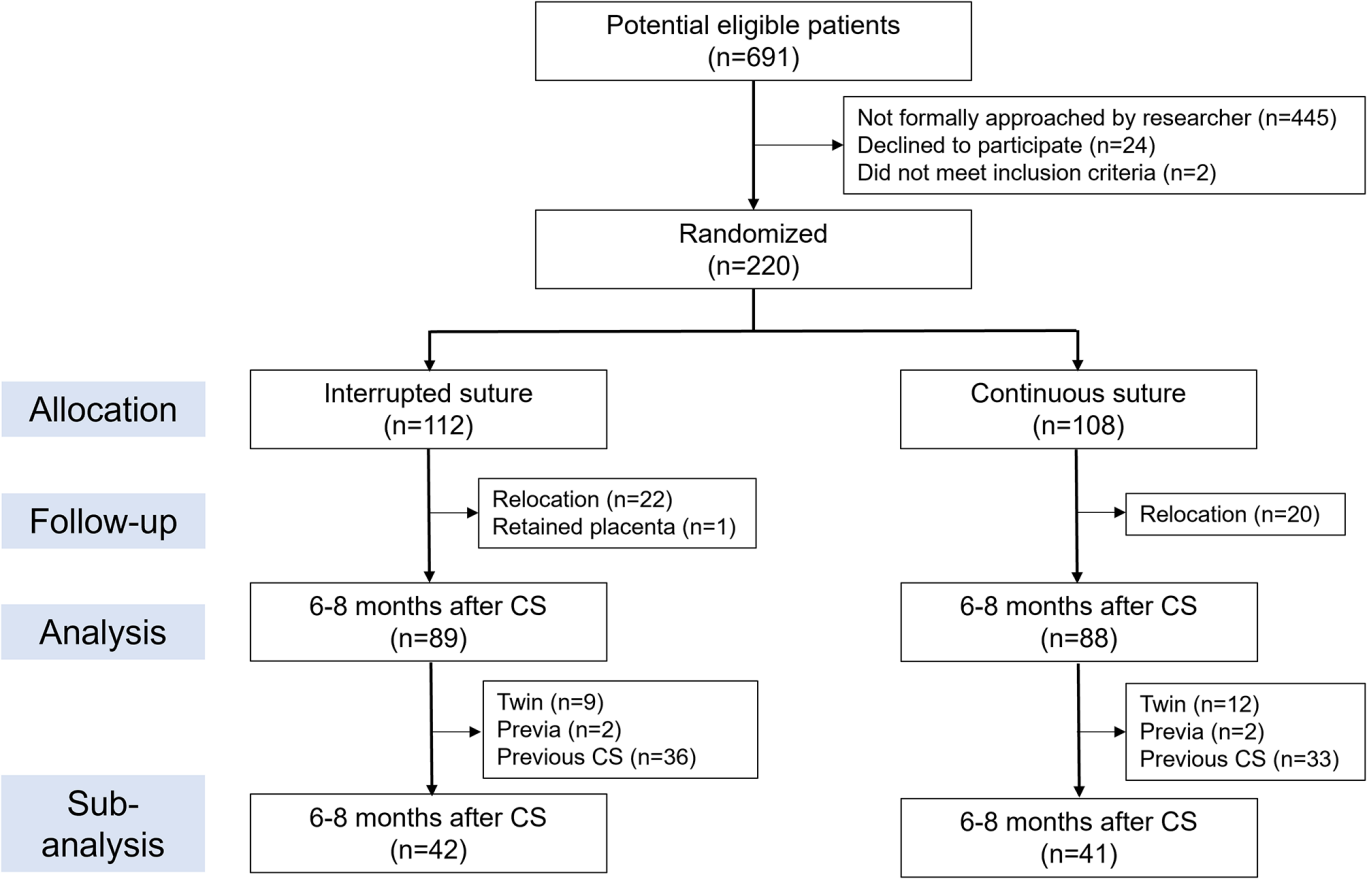
Flowchart of the study

All participants enrolled in this study provided written informed consent before participating. Before CS, the participants were randomly assigned to the two-layer interrupted suture group (interrupted group) or the two-layer unlocked continuous suture group (continuous group) (Fig. 1). The person controlling participant assignment provided the registration number in Excel to randomly assign the participants to interrupted and continuous groups in advance. The allocation was made with 20 cases in each block, resulting in 11 blocks. A code sheet was created for 220 cases, and the allocation code table was stored appropriately by the person in charge of allocation. Although the operator was informed of the suturing method shortly before CS, participants were not informed of their allocation.

### Intervention

**Fig. 2:**
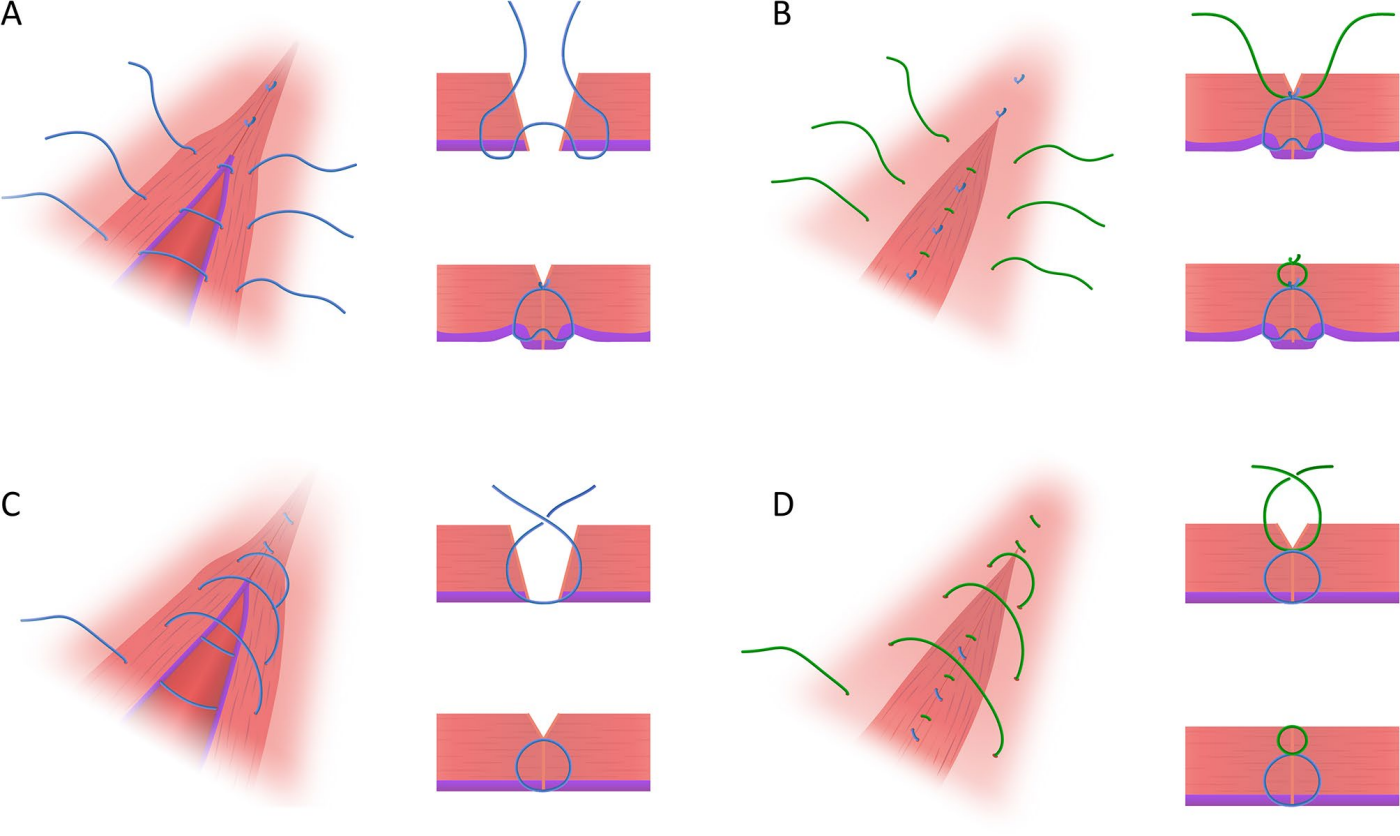
Schema of the suture technique in the interrupted (**A**, **B**) and continuous groups (**C**, **D**). (**A**) Interrupted suture for first layer using modified Gambee suture technique (**B**) Interrupted suture for second layer (**C**) Un-lock continuous suture for first layer (**D**) Un-lock continuous suture for second layer

The synthetic absorbable multifilament thread was used in both groups (Coated VICRYL^®^ [polyglactin 910], size 0, Ethicon Inc., a Johnson & Johnson Company, Somerville, NJ, USA). In both groups, two single interrupted sutures were applied to both ends of the incision before the first suture layer was applied. Before suturing the uterine wound, the myometrial thickness at the center of the uterine wound on the fundal and cervical sides was measured using a sterilized ruler. The first layer was sutured in the interrupted group using modified Gambee sutures (Fig. 2A) [12]. Next, the second layer was sutured between the knots of the first layer (Fig. 2B). In the continuous group, the first layer, including the slight decidua, was sutured (Fig. 2C). Needles for the second layer were inserted between the first-layer threads (Fig. 2D). After suturing the myometrium, an absorbable anti-adhesion barrier (INTERCEED^®^, Ethicon, Johnson & Johnson Company) was attached to the vesicouterine pouch in both groups. Subsequently, the ventral peritoneum, fascia, and dermis were closed. Before starting the trial, the surgeons received a lecture on surgical techniques and were limited to certified specialists from the Japan Society of Obstetrics and Gynecology.

### Outcomes

**Fig. 3:**
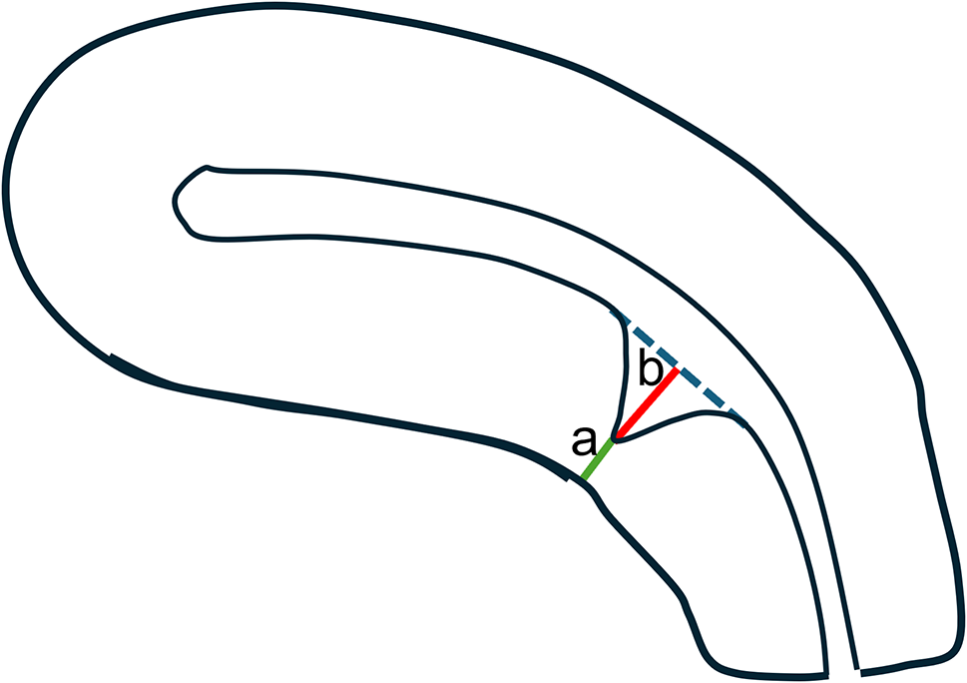
Evaluation of the healing ratio (HR) in cesarean scar defects. (**a**) Residual myometrial thickness (**b**) Depth of the cesarean scar defect. However, these measurements do not include the endometrium. HR = a/a + b

The primary outcome was the RMT at 6–8 months post-CS. Sonohysterography was conducted by the same examiner throughout the study to measure the RMT. One examiner saved the images, and the other examiner, blinded to the allocation, measured the RMT. The secondary outcome was the rate of severe CSD, defined as a healing ratio (HR) < 0.5, as described in a previous study [11]. The HR was calculated as RMT / (RMT + CSD depth) (Fig. 3). In other words, severe CSD means a loss of over 50% of the myometrium. In addition, we checked whether the uterus was retroverted using TVU. A sub-analysis was then performed, excluding twin pregnancies, previous sections, and placenta previa (Fig. 1), and similar primary and secondary evaluations were performed.

### Sample size

Based on a previous report, the estimated difference in RMT between the two groups was 0.85 mm, and the standard deviation (SD) was 1.73 [13]. The sample size was calculated using an alpha and a beta error of 0.05 and 0.90, respectively. As a result, the required sample size per group was 88, and 176 patients were required. However, women often return to their hometowns to give birth in Japan; therefore, it is expected that some of these participants will move and drop out 6–8 months after delivery. Therefore, we concluded that 220 patients were required, assuming a dropout rate of 20%.

### Statistical analysis

Statistical analyses were performed using GraphPad Prism version 9 (GraphPad Software Inc., San Diego, CA, USA). The normality of the distribution of continuous variables was assessed using the D’ Agostino–Pearson test. Normally distributed data was described as mean $\:\pm\:\:$SD, whereas non-normally distributed data are presented as median (interquartile range). Comparisons between both groups were performed using an unpaired 2-tailed t-test for normally distributed variables and Mann–Whitney U tests for nonparametric data. Categorical data were analyzed using Fisher exact test. A multivariate regression analysis was conducted to identify the factors associated with severe CSD. JMP^®^ 15 (SAS Institute Inc., Cary, NC, USA) was used for multivariate analysis. Statistical significance was set at *p* < 0.05.

## Results

Of the 220 participants, 112 cases were assigned to the interrupted group, and 108 were assigned to the continuous group between May 2020 and April 2023 (Fig. 1). The last follow-up date was October 2023. In the interrupted group, a case of retained placenta and intrauterine intervention performed post-CS was excluded from this study. We compared 89 participants in the interrupted group and 88 participants in the continuous group after excluding those who relocated after birth. The baseline characteristics are shown in Table 1. No significant differences were observed between the two groups regarding any of the items.

**Table 1:** Baseline characteristics of participants in both groups

	Interrupted (*n* = 89)	Continuous (*n* = 88)	*p*-value
Age, y	34 (30–38)	34 (32–38)	0.513
BMI, kg/m^2^	23 (21–26)	24 (22–26)	0.805
Gravidity	2 (1–3)	2 (1–3)	0.124
Parity	1 (0–1)	0 (0–1)	0.183
Number of CS			
0	53	55	0.759
1	31	28	0.750
2	5	5	> 0.999
Indication of CS			
Previous CS	36	33	0.646
Breech presentation	19	14	0.441
Twin	9	12	0.495
Arrest of labor	6	3	0.496
NRFS	5	8	0.405
History of myomectomy	5	9	0.280
Preeclampsia	4	1	0.368
Fetal anomaly	3	3	> 0.999
Previa	2	3	0.682
Maternal complication	0	2	0.246
Scheduled CS	54	62	0.206
Emergency CS	35	26	0.206
Dilatation of Os at CS			
0–4 cm	85	87	0.368
5 cm	4	1	0.368

BMI, body mass index; CS, cesarean section; NRFS, non-reassuring fetal status

**Fig. 4:**
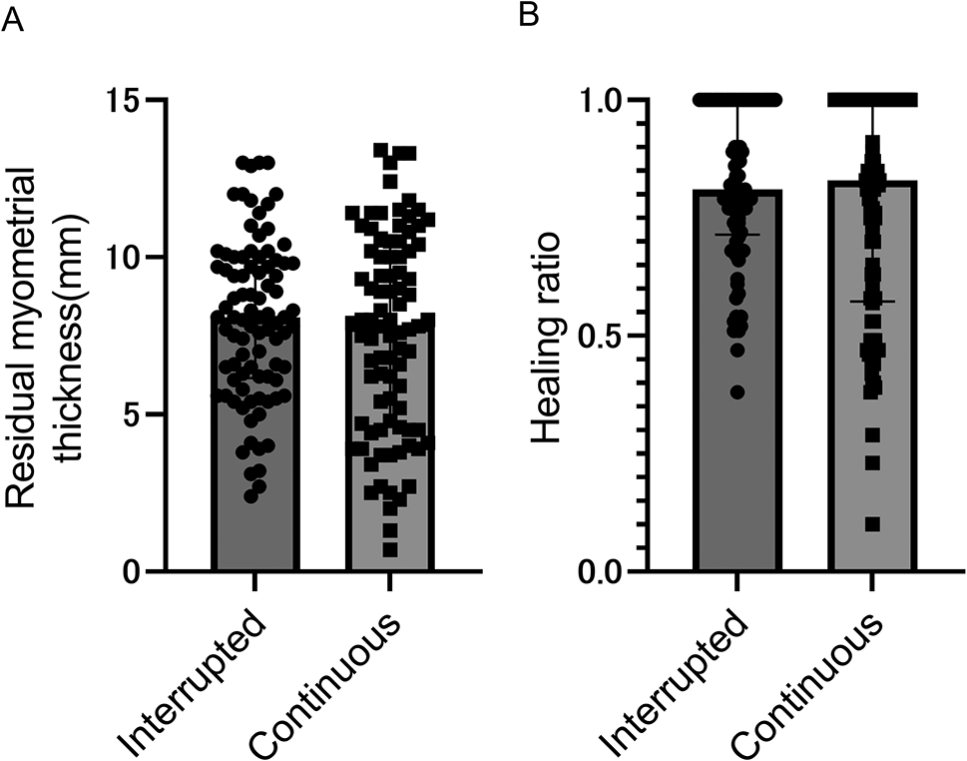
Results of residual myometrial thickness (**A**) and healing ratio (**B**) 6–8 months after CS. Scatter plot with bar indicates median with interquartile range. CS, cesarean section

Regarding surgery and short-term prognosis, the number of additional stitches required to stop the bleeding was significantly higher in the interrupted group than in the continuous group (Table 2). However, no significant differences were observed between the two groups regarding other parameters, including the total operative time and total blood loss (Table 2). RMT values 6–8 months after CS were 8.1 (interquartile range, 6.2–9.9) mm in the interrupted group and 7.9 (interquartile range, 4.6–10.3) mm in the continuous group (Fig. 4A), demonstrating no significant differences in RMT as the primary outcome for all participants. The HR values 6–8 months after CS were 0.81 (interquartile range, 0.71–1.00) and 0.83 (interquartile range 0.57–1.00) in the interrupted and continuous groups, respectively, with no significant difference (Fig. 4B). However, the incidence of severe CSD was significantly lower in the interrupted group than in the continuous group (Table 3).

**Table 2:** Surgery information and short-term prognosis

	Interrupted (*n* = 89)	Continuous (*n* = 88)	*p*-value
Gestational week at CS, wk	37 (37–38)	37 (36–38)	0.766
Weight of birth, g	2702 (2445–2981)	2661 (2229–2998)	0.467
Myometrial thickness (fundal side), mm	10 (9–12)	10 (9–12)	0.544
Myometrial thickness (cervical side), mm	8 (7–11)	9 (7–11)	0.620
Myometrial thickness difference, mm	2 (0–3)	1 (0.5–2)	0.540
Number of additional stiches to stop the bleeding	1 (0–2)	0 (0–1)	0.002
Total operative time, min	59 (54–69)	58 (49–67)	0.122
Total blood loss, mL	950 (697–1333)	886 (693–1293)	0.694
Presence of postoperative fever	9 (10%)	4 (4.8%)	0.249
CRP value on the 1st day after CS	6.7 (4.4–8.9)	6.0 (4.1–8.5)	0.442
Postoperative hospitalization days for CS	6.0 (6.0–6.0)	6.0 (6.0–6.0)	0.731

CRP, C-reactive protein; CS, cesarean section

**Table 3:** Sonohysterography findings 6–8 months after CS

	Interrupted (*n* = 89)	Continuous (*n* = 88)	*p*-value
Retroverted uterus	29 (33%)	35 (40%)	0.350
Presence of CSD	37 (42%)	41 (47%)	0.546
Presence of sever CSD	2 (2%)	19 (22%)	< 0.0001

CSD, cesarean scar defect; CS, cesarean section

In the sub-analysis limited to primary CS, no differences were noted in the patient background (Table 4), and no significant difference was observed in the surgical condition or short-term prognosis, except for the number of additional stitches required to stop the bleeding (Table 5). Notably, no significant differences were observed in RMT, HR, frequency of retroverted uterus, and CSD. However, the incidence of severe CSD in the interrupted group was significantly lower than that in the continuous group (Table 6).

**Table 4:** Baseline characteristics of participants in the sub-analysis

	Interrupted (*n* = 42)	Continuous (*n* = 40)	*p* value
Age, y	33 (29–38)	34 (31–38)	0.436
BMI, kg/m^2^	23 (21–25)	24 (22–26)	0.159
Gravidity	1 (1–2)	1 (1–2)	0.280
Parity	0 (0–0)	0 (0–0)	0.259
Number of CS			
0	42	40	
1	0	0	
2	0	0	
Indication of CS			
Previous CS	0	0	
Breech presentation	19	14	0.4408
Twin	0	0	
Arrest of labor	5	3	0.720
NRFS	5	8	0.405
History of myomectomy	6	9	0.433
Preeclampsia	4	1	0.368
Fetal anomaly	3	3	> 0.999
Previa	0	0	
Maternal complication	0	2	0.235
Scheduled CS	17	25	0.051
Emergency CS	25	15	0.051
Dilatation of Os at CS			
0–4 cm	38	39	0.360
5 cm	4	1	0.360

BMI, body mass index; CS, cesarean section; NRFS, non-reassuring fetal status

**Table 5:** Surgery information and short-term prognosis in the sub-analysis

	Interrupted (*n* = 42)	Continuous (*n* = 40)	*p*-value
Gestational week at CS, weeks	37 (36–38)	37 (37–38)	0.762
Weight of birth, g	2628 (2294–3054)	2824 (2441–3064)	0.626
Myometrial thickness (fundal side), mm	11 (10–13)	11 (9–12)	0.532
Myometrial thickness (cervical side), mm	10 (8–11)	10 (7–12)	0.990
Myometrial thickness difference, mm	2 (0–3)	1 (0–2)	0.486
Number of additional stiches	1 (0–2)	0 (0–1)	0.072
Total operative time, min	57 (51–66)	55 (45–62)	0.235
Total blood loss, mL	865 (512–1161)	772 (625–1068)	0.610
Presence of postoperative fever	3 (7%)	3 (8%)	0.249
CRP value on the 1st day after CS	7.0 (4.1–8.4)	5.5 (4.1–8.4)	0.198
Postoperative hospitalization days for CS	6.0 (6.0–6.0)	6.0 (6.0–6.0)	0.789

CS, cesarean section; CRP, C-reactive protein

**Table 6:** Sonohysterography findings 6–8 months after CS in the sub-analysis

	Interrupted (*n* = 42)	Continuous (*n* = 40)	*p*-value
RMT (mm), median (IQR)	8.6(6.8–10.5)	9.2(6.0–11.0)	0.705
Healing ratio, median (IQR)	0.95 (0.78–1.00)	0.90 (0.71–1.00)	0.362
Retroverted uterus	14 (33%)	15 (38%)	0.818
Presence of CSD	11 (26%)	15 (38%)	0.344
Presence of severe CSD	0 (0%)	7 (18%)	0.0117

RMT, residual myometrial thickness; IQR, interquartile range; CSD, cesarean scar defect; CS, cesarean section

A multivariate logistic regression analysis was performed to determine the factors contributing to severe CSD (Table 7). The analysis revealed that interrupted sutures significantly decreased the incidence of severe CSD (odds ratio: 0.04, 95%CI: 0.005–0.261, *p* = 0.0011) compared with continuous sutures. In addition, more significant differences in myometrial thickness and retroverted uterus increased severe CSD development (odds ratio: 1.64, 95%CI: 1.179–2.458, *p* = 0.008 and odds ratio: 3.57, 95%CI: 1.103–11.576, *p* = 0.034, respectively).

**Table 7:** Results of multivariate logistic regression analysis for the development of severe CSD

Value	Odd ratio	95% CI	*p* value
Age	0.98	0.883–1.076	0.632
BMI	1.04	0.948–1.176	0.480
Dilatation of Os	1.08	0.725–1.608	0.702
Number of additional stiches	1.20	0.666–2.022	0.515
Total operative time	1.00	0.956–1.042	0.978
Total blood loss	1.00	0.999–1.001	0.673
Interrupted suture	0.04	0.005–0.261	0.001
Myometrial thickness difference	1.64	1.179–2.458	0.008
Retroverted uterus 6–8 months after CS	3.57	1.103–11.576	0.034

BMI, body mass index; CS, cesarean section

## Discussion

### Main findings

This randomized controlled trial revealed that two-layer interrupted sutures prevent severe CSD formation compared with two-layer unlocked continuous sutures. Furthermore, we demonstrated that the difference in myometrial thickness at the time of suturing and retroversion of the uterus 6–8 months post-CS was significantly associated with the incidence of severe CSD.

### Interpretation

Notably, there are various reports on suture research for CS [3–6], but none have investigated the association between two-layer interrupted sutures and CSD formation. A previous study demonstrated that the size of the CSD after a single-layer interrupted suture was significantly smaller than that after a single-layer continuous suture [7]. The paper discussed that interrupted sutures might be linked to maintaining blood flow in the wound compared to continuous sutures. Tekelioğlu et al. demonstrated that the more significant difference in myometrium thickness between the fundal and cervical sides was significantly associated with the incidence of CSD [13]. Regarding a retroverted uterus, Ofili-Yebovi et al. [11] revealed that a retroflexed uterus had a strong association with the incidence of CSD. The relationship between uterine retroversion and CSD has been described in several recent reviews on CSD formation [14, 15]. Our findings were consistent with these previous reports [7, 13–15].

The main analysis included patients who had a previous cesarean section. Therefore, it is possible that CSD had already existed before this cesarean section. Therefore, a sub-analysis was conducted that was limited to primary CS. The sub-analysis revealed that severe CSD was not observed in primary CS in the two-layer interrupted group. The result demonstrated that interrupted sutures contributed to the prevention of severe CSD, regardless of a history of CS.

Although CSD was defined as an indentation at the site of the CS scar with a depth of at least 2 mm, not all cases of CSD are symptomatic. Considering previous studies, the severe CSD defined in this study is appropriate as a condition for presenting symptoms [9–11]. A recent systematic review and meta-analysis also showed that patients with larger CSD experienced more bleeding symptoms than those with smaller CSD [16]. Therefore, we believe that preventing severe CSD formation helps to maintain the quality of life in women with a history of CS. Regarding the timing of CSD evaluation, Jiang et al. [17] reported that 6 weeks after CS was too early, but 6–8 months after CS was valid. According to this report, if the timing of the CSD evaluation is 6 weeks, there is a possibility of misdiagnosis or overdiagnosis; however, no change occurs in the size of the CSD at 6 months and 1 year after CS. Hence, we believe that there is validity to the timing of the CSD evaluation in this study.

This study demonstrated no significant differences in RMT values, but there was a significant difference in the frequency of severe CSD. This result may seem surprising; however, as the spread of the dot plot in Fig. 3 shows, there are differences in the variation of RMT and HR results. This result suggests a more considerable HR variation in the continuous group than in the interrupted group. This result is similar to that of previous report [7], and we believe that this extensive variation results in an increased incidence of severe CSD in the continuous suture group. This significant variation could be caused by the instability or uncertainty of continuous sutures, but this study could not clarify the cause.

The significant correlation between the difference in myometrial thickness at the time of suturing and severe CSD development may indicate the importance of the uterine incision level in CS. Vikhareva et al. [18] demonstrated that a low-incision group had a significantly higher rate of significant scar defects than a high-incision group. We believe that these results suggest that a high incision may have contributed to preventing the formation of severe CSD by reducing the difference in myometrial thickness after uterine incision. Thus, further study will be necessary to evaluate the association between the hysterotomy level and differences in myometrial thickness after delivery of the placenta at CS. In other words, appropriate suture technique alone may not prevent severe CSD because many factors contribute to CSD formation, not only the hysterotomy level, but the presence of pelvic adhesion, obstetrical complications, and cervical dilation [2].

Outcomes other than CSD should also be considered by obstetricians. Sumigama et al. [19] reported that continuous sutures were significantly more likely to result in placenta previa in subsequent pregnancies than interrupted sutures. Therefore, interrupted suture may contribute not only to the prevention of CSD formation, but also to the prevention of placenta previa in the patient’s subsequent pregnancy.

### Strengths and limitations

To the best of our knowledge, this is the first report comparing two-layer interrupted sutures and two-layer continuous sutures from the viewpoint of preventing CSD formation. The strength of this study is that it was a prospective randomized controlled study conducted at a single institution. Because this study compared surgical techniques, the surgeons’ skill differences may have affected the results. Therefore, before starting the research, surgical techniques were confirmed within the same facility to eliminate possible differences in skill between surgeons.

Regarding limitations, although no difference was observed in the background between the two groups, the participants in this study included various cases, indications for CS, the number of CS, scheduled and emergency CS. However, no difference was observed in the evaluation of patient background between both groups. Second, there are also limitations specific to Japan. The post-CS hospital stay in Japan is long, lasting about 1 week. In addition, patients usually like to return to their hometown temporarily for delivery. Therefore, recruiting participants who had not moved 6 months after giving birth was challenging. This phenomenon leads to high dropout rates. Lastly, the present study did not reveal any association between the suture method and clinical presentation, as CSD was assessed at 6–8 months after CS and menstruation had not resumed or was not yet cyclic. Differences in symptoms between the groups should be evaluated in the future.

## Conclusions

Two-layer interrupted sutures reduce the incidence of severe CSD compared with two-layer continuous sutures during CS. These findings may contribute to the prevention of various symptoms of CSD in postpartum women who undergo CS.

## Electronic supplementary material

Below is the link to the electronic supplementary material.

Supplementary Material 1

## Data Availability

The data supporting this study’s findings are available on request from the corresponding author. The data are not publicly available due to privacy or ethical restrictions.

## Abbreviations

CS: Cesarean section
CSD: Cesarean scar defect
RMT: Residual myometrial thickness
SD: Standard deviation
TVU: Transvaginal ultrasonography
OR: Odds ratio
CI: Confidence interval
